# Murine Cytomegalovirus Infection Induces Susceptibility to EAE in Resistant BALB/c Mice

**DOI:** 10.3389/fimmu.2017.00192

**Published:** 2017-02-27

**Authors:** Jelena Milovanovic, Branka Popovic, Marija Milovanovic, Daria Kvestak, Aleksandar Arsenijevic, Bojana Stojanovic, Irena Tanaskovic, Astrid Krmpotic, Nebojsa Arsenijevic, Stipan Jonjic, Miodrag L. Lukic

**Affiliations:** ^1^Center for Molecular Medicine and Stem Cell Research, Faculty of Medical Sciences, University of Kragujevac, Kragujevac, Serbia; ^2^Faculty of Medical Sciences, Institute of Histology, University of Kragujevac, Kragujevac, Serbia; ^3^Center for Proteomics, Faculty of Medicine, Department for Histology and Embryology, University of Rijeka, Rijeka, Croatia; ^4^Faculty of Medical Sciences, Institute of Pathophysiology, University of Kragujevac, Kragujevac, Serbia

**Keywords:** experimental autoimmune encephalomyelitis, BALB/c mice, murine cytomegalovirus infection, antigen-presenting cells, T cells

## Abstract

In contrast to C57BL/6 mice, BALB/c mice are relatively resistant to the induction of experimental autoimmune encephalomyelitis (EAE) after challenge with MOG_35–55_ peptide. Here, we provide the first evidence that infection with murine cytomegalovirus (MCMV) in adulthood abrogates this resistance. Infected BALB/c mice developed clinical and histological signs similar to those seen in susceptible C57BL/6 mice. In addition to CD4^+^ cells, large proportion of cells in the infiltrate of diseased BALB/c mice was CD8^+^, similar with findings in multiple sclerosis. CD8^+^ cells that responded to *ex vivo* restimulation with MOG_35–55_ were not specific for viral epitopes pp89 and m164. MCMV infection favors proinflammatory type of dendritic cells (CD86^+^CD40^+^CD11c^+^) in the peripheral lymph organs, M1 type of microglia in central nervous system, and increases development of Th1/Th17 encephalitogenic cells. This study indicates that MCMV may enhance autoimmune neuropathology and abrogate inherent resistance to EAE in mouse strain by enhancing proinflammatory phenotype of antigen-presenting cells, Th1/Th17, and CD8 response to MOG_35–55_.

## Introduction

Multiple sclerosis (MS) is a chronic inflammatory, demyelinating disease of the central nervous system (CNS) with axonal injury, characterized by varying clinical course, pathology, and inflammatory patterns (1). It develops in susceptible hosts after interaction with environmental factors which trigger the disease by promoting the activation of myelin-specific T cells that normally circulate in the peripheral lymph organs of all individuals (2). It has been suggested that some infectious agents, in particular viruses, may be potential triggers of MS (2–4). Among different infective agents, Epstein–Barr virus (EBV) has been mostly associated with increased MS risk (5). Recently, it has been shown increased CD8^+^ T cell response to EBV lytic antigens in active MS and also in relapses (6). Infection with murine gamma herpesvirus 68 (γHV-68), the murine homolog to EBV, polarizes the adaptive immune response and heightens CNS pathology following experimental autoimmune encephalomyelitis (EAE) induction and likely, influences MS pathogenesis (7).

Experimental autoimmune encephalomyelitis is the experimental model of MS, induced in susceptible animals by active immunization with myelin antigens mixed with adjuvant (8). Immunized mice develop ascending paralysis with CD4^+^ T cells and macrophages in infiltrations in the white matter of the spinal cord, and with minimal brain inflammation in the majority of experimental models. However, in MS, the vast majority of myelin lesions are found within the brain parenchyma with infiltrations that contain equivalent numbers of CD8^+^ T and CD4^+^ T cells (9, 10). Despite these differences, EAE is considered as a valuable tool for research of MS pathogenesis. Moreover, several therapeutics that are now being used to treat MS were developed in EAE (11). BALB/c mice are found partially or completely resistant to the induction of EAE with encephalitogenic peptide, myelin oligodendrocyte glycoprotein (MOG_35–55_).

Cytomegalovirus (CMV) classified within the *Betaherpesvirinae* subfamily establishes life-long latent infections in 70–100% of the human population (12). After a primary infection of fibroblasts, epithelial, endothelial, and smooth muscle cells (6), mostly asymptomatic in the immunocompetent host, CMV persists in myeloid precursor cells (7). During latency periodic asymptomatic reactivations occur (13). CMV contains a large number of latent and lytic genes, many of which code proteins that have the role in immunoregulation (5). When monocytes that carry CMV enter visceral parenchyma and differentiate into macrophages and myeloid dendritic cells, virus reactivates and through expression of different genes can modulate the immune response of the host (14).

Data on the role of CMV infection in etiopathogenesis of MS are controversial. CMV has been found in demyelinating plaques and the cerebrospinal fluid of MS patients (15) and causes demyelination mainly in the CNS of immunocompromised hosts (16). Further, enhancement of numbers of EBV and CMV-specific CD8^+^ T cells among T cells in chronic inflammatory lesions of brain of MS patients was reported (17). Several studies involving human subjects indicate correlation between CMV infection and MS development, greater rate of relapses and greater brain atrophy (18–20). Other studies indicate that CMV seropositivity is associated with a decreased MS risk and predicts a better clinical and radiological outcome in MS patient (21), suggesting a protective effect of CMV on autoimmune neuropathology (22). Furthermore, CMV encodes multiple factors that trigger immunomodulatory or evasion mechanisms, which can decrease the immune response in MS patients (23, 24).

We have recently shown that deletion of an immunoregulatory pathway, IL-33/ST2 axis, may enhance susceptibility to EAE in resistant BALB/c strain (25, 26). The present study was done with the aim to explore whether infection with murine CMV (MCMV) create “fertile field” (27, 28) that facilitates the expansion and activation of encephalitogenic cells leading to autoimmune disease of CNS.

Here, we show that MCMV infected and MOG_35–55_ immunized BALB/c mice develop very pronounced neuroinflammation with extensive infiltrations in brain and spinal cord parenchyma containing large proportion of CD8^+^ cells in infiltrates in addition to accentuation of Th1 and Th17 immune response and skewing microglia to M1 phenotype. Our results are compatible with the notion that MCMV abrogates inherent resistance of BALB/c mice to EAE induction with MOG_35–55_ peptide through enhancement of inflammatory dendritic cells in the periphery, M1 type of microglia and recruitment of MOG_35–55_ responsive CD8^+^ T cells in the CNS. Thus, CMV-induced inflammatory environment may enhance autoimmunity in CNS.

## Materials and Methods

### Infection, Induction, and Scoring of EAE

Female 8-week-old BALB/c mice were used throughout this study. Mice were infected subcutaneously (footped) with 10^5^ plaque-forming units of tissue culture MCMV, strain MW97.01 (29). EAE was induced 10 days after infection by subcutaneous administration of 200 µL suspension at two sites over the hind flanks. Depletion of CD4^+^ lymphocytes, where indicated, was performed with intraperitoneal injection of 100 µg of anti-CD4 mAb, 1 day prior to and 5 days after MOG_35–55_ immunization. The suspension consisted of 300 µg MOG_35–55_ peptide (Sigma-Aldrich, Germany) in 100 µL of PBS, emulsified with 100 µL complete Freund’s adjuvant (Sigma-Aldrich, Germany) with 0.7 mg heat-inactivated *Mycobacterium tuberculosis* (strain H37 RA; Difco Laboratories, Detroit, MI, USA). Each mouse was immediately thereafter, injected intraperitoneally and 48 h later with 300 ng pertussis toxin (List Biological Laboratories, Campbell, CA, USA) in 100 µL 0.9% NaCl. Clinical signs of EAE were assessed daily by the following scoring system: grade 0, no signs; grade 1, paralyzed tail; grade 2, ataxic; grade 2.5, one hind leg paralyzed; grade 3, both hind legs paralyzed; grade 3.5, three legs paralyzed; grade 4, both hind legs and front limbs completely paralyzed; grade 5, moribund as previously described (30, 31). Mice were monitored daily with fluid administration and mashed chow on the base of cages for all mice displaying a clinical score of 3. Mice were maintained in our animal facilities in a temperature-controlled environment with a 12-h light/12-h dark cycle and were administered standard laboratory food and water *ad libitum*. All experiments were approved by and conducted in accordance with the Guidelines of the Animal Ethics Committee of Faculty of Medical Sciences, University of Kragujevac, Serbia. Endangered animal species were not used in this study.

### Isolation of Mononuclear Cells from CNS and Lymph Nodes

At day 15 post-EAE induction (mean clinical score of 3 for MCMV EAE mice), mice were perfused with PBS, and brain and spinal cord were carefully removed. The mononuclear cells from CNS were isolated as described previously (25). Briefly, the brains and spinal cords were homogenized in RPMI 1640 (Sigma-Aldrich) with 10% FBS and 1 mg/mL collagenase type I (Sigma-Aldrich) and incubated at 37°C for 60 min. After digestion, the tissue was passed through a 40-µm mesh, pelleted, resuspended in 10 mL 30% Percoll (Sigma-Aldrich), overlaid onto 5 mL 70% Percoll, and centrifuged at 390 *g* for 20 min. The myelin layer was removed, and the mononuclear cells accumulated in the intermediate phase were collected, washed twice in PBS, and resuspended in RPMI 1640 containing 10% FBS. Total cell numbers were determined by counting on a hemocytometer, and viability was assessed by trypan blue exclusion. Lymph nodes were minced in RPMI 1640 (Sigma-Aldrich) and forced gently through 40-µm cell-strainer nylon mesh (Falcon) using a sterile syringe plunger and centrifuged at 400 *g* for 5 min. Pellet from lymph nodes was resuspended in RPMI 1640 containing 10% FBS.

### Flow Cytometry

Single-cell suspensions of brain and spinal cord tissue were prepared according to standard protocols. For cytofluorometry, following antibodies were used: CD4, CD8, CD45, CCR6, CXCR3, TCRβ, CD11c, CD11b, CD49b, CCR2, CD86, T-bet, RORγt, IL-17, IFN-γ, TNF-α, and IL-12 with conjugated fluorochromes (BD Biosciences). Antibodies were incubated with cells in PBS with 2% FBS for 30 min at 4°C, and then cells were analyzed. For intracellular staining of cytokines, cells were stimulated for 4 h in RPMI 1640 containing 10% FBS (Gibco), 10 ng/mL phorbol 12-myristate 13-acetate (Sigma-Aldrich), and 500 ng/mL ionomycin (Sigma-Aldrich) with addition of Brefeldin A (BD Biosciences). Antibodies for the cell surface markers were added to the cells in PBS with 2% FBS for 30 min on ice. After wash, cells were resuspended in Cytofix/Cytoperm buffer (BD Biosciences) for 20 min on ice, washed twice, and incubated with Abs for intracellular antigens (cytokines) in Perm buffer (30 min, on ice). For staining of transcriptional factors, unstimulated cells were used. Data were acquired using FACSCalibur (BD Biosciences) and analyzed with FlowJo software (Tree Star).

### Tetramer Staining

Immune cells were isolated as described above. Cells isolated from CNS were incubated with H-2L(d)/IE-1/pp89 (168–176 YPHFMPTNL) and H-2D(d)/m164 (257–265 AGPPRYSRI) tetramers provided by NIH tetramer core facility. Cells stained with tetramer and anti-CD8 and anti-CD3 antibodies were incubated for 30 min at room temperature and then washed. Data were acquired using a FACSCalibur (BD Biosciences) and analyzed with FlowJo software (Tree Star).

### Immunohistochemistry and Evaluation of Brain and Spinal Cord Pathology

Brains and spinal cords were fixed in 4% buffered formalin fixative overnight. Paraffin wax embedded sections (5 µm) were stained with hematoxylin and eosin and CD3 (ab699; Abcam) immunohistochemical staining. The slides were analyzed on light microscope (BX51; Olympus), and digital images were acquired by digital camera. The level of infiltration was graded using the following score: 0, no inflammatory cells; 1, a few scattered inflammatory cells; 2, organization of inflammatory infiltrates into perivascular cuffs; 3, extensive perivascular cuffing with extension into adjacent subarachnoid space and CNS parenchyma, and 4, extensive perivascular cuffing with increasing subarachnoid and parenchymal inflammation (32). Slides were analyzed on Olympus BX51 microscope, and digital images were acquired by Olympus digital camera (DP71).

### Interferon-γ Assay

Mononuclear cells isolated from CNS, 10^5^ in 100 µL complete media were put on 96-well plate in duplicates, and 100 µL of media, or MOG_35–55_ (1 μg/well) were added. After incubation for 1 h on 37°C, 0.2 µL of Brefeldin A in 10 µL of medium was added to each well and incubated for 4 h on 37°C. Cells were washed and then incubated with anti-CD8 and anti-CD4 antibodies on +4°C for 15 min. After washing, cells were resuspended in Cytofix/Cytoperm buffer for 30 min on ice, washed twice, and incubated with anti-IFNγ antibodies diluted in Perm wash buffer (30 min, on ice) and resuspended in FACS media.

### Statistical Analysis

All statistics were carried out using SPSS 18.0 for Windows software. Results were analyzed using the Student’s *t*-test or Mann–Whitney test and ANOVA or Kruskal–Wallis. Data in this study were expressed as the mean + SEM. Values of *P* < 0.05 were considered significant.

## Results

### MCMV Infection in Adult Life Abrogates Resistance to EAE in BALB/c Mice

**Figure 1:**
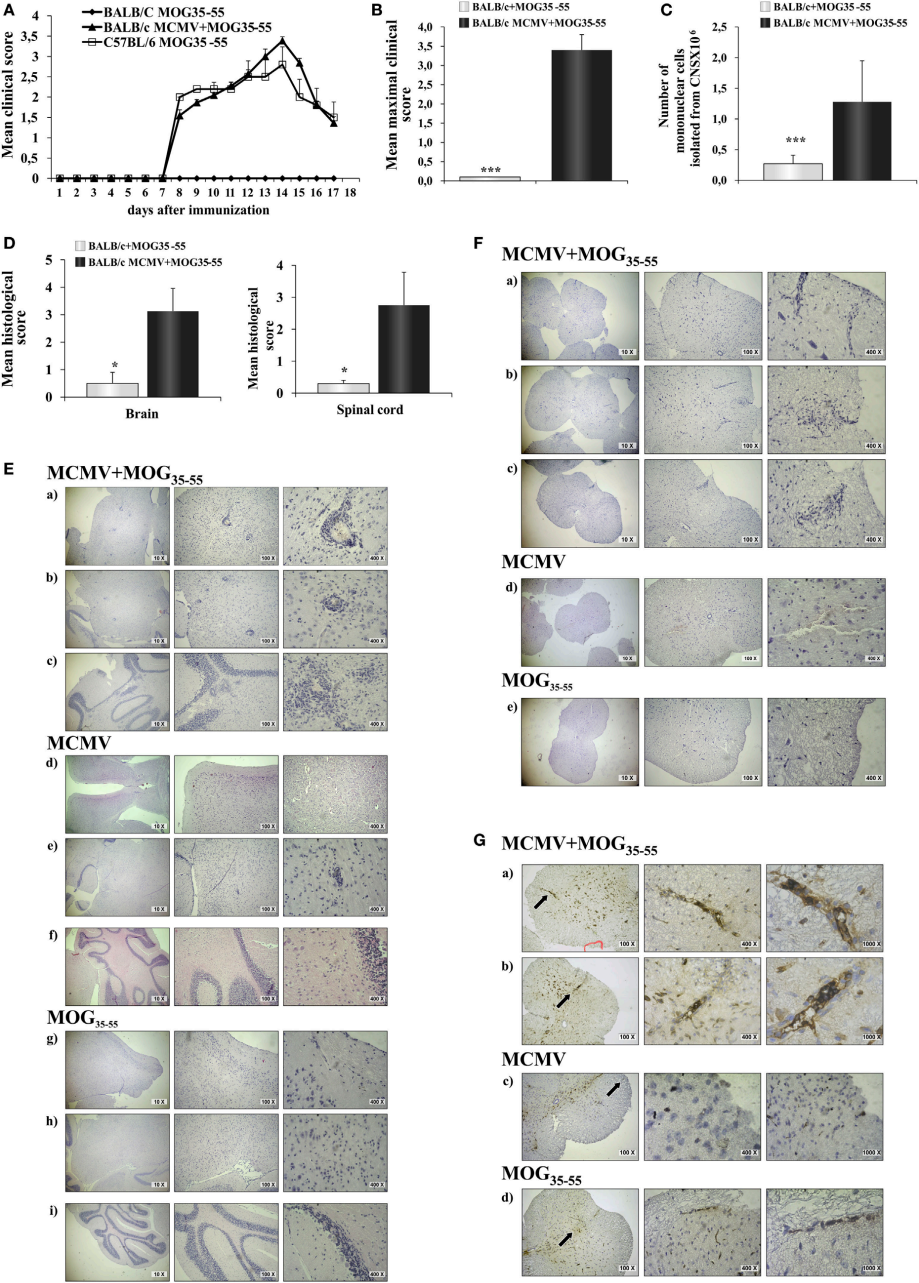
**BALB/c mice infected with murine cytomegalovirus (MCMV) and immunized with MOG_35–55_ develop experimental autoimmune encephalomyelitis (EAE)**. Eight-week-old BALB/c mice were infected (foot-pad injection) with MCMV and 10 days after were immunized with MOG_35–55_ peptide in CFA and pertussis toxin (BALB/c MCMV + MOG_35–55_). Control mice C57BL/6 and BALB/c were immunized with MOG_35–55_ peptide in CFA and pertussis toxin without previous infection (C57BL/6 MOG_35–55_ and BALB/c MOG_35–55_). **(A)** EAE scores up to day 15 post-EAE induction (four separate experiments, *n* = 29/group). **(B)** Mean maximal clinical score up to day 15 post-EAE induction (four separate experiments, *n* = 29/group). **(C)** The mean value of the mononuclear cells isolated from central nervous system (CNS) of BALB/c MCMV + MOG_35–55_ and BALB/c MOG_35–55_ mice (three independent experiments, *n* = 24/group). **(D)** Mean histological scores were calculated from a total of five sections per group (two separate experiments, *n* = 8/group). **(E)** The representative images of brain cortex (a), brain stem (b), cerebellum (c) of BALB/c MCMV + MOG_35–55_; brain cortex (d), brain stem (e), cerebellum (f) of BALB/c MCMV-infected mice (BALB/c MCMV); and brain cortex (g), brain stem (h), cerebellum (i) of BALB/c MOG_35–55_ mice. **(F)** The representative images of spinal cords of BALB/c MCMV + MOG_35–55_ (a–c); BALB/c MCMV (d); and BALB/c MOG_35–55_ mice (e). **(G)** Representative sections of CD3 spinal cord immunohistochemistry of BALB/c MCMV + MOG_35–55_ (a,b); BALB/c MCMV (c); and BALB/c MOG_35–55_ mice (d), arrows in left panels indicate the area presented in magnified sections in right panels. All pictures are representative of two separate experiments (*n* = 16/group). Data were analyzed by Student’s *t*-test and presented as mean + SE: *P < 0.05 and ***P < 0.001.

BALB/c mice immunized with MOG_35–55_ did not develop clinical signs of EAE, while BALB/c mice infected with MCMV 8 weeks after birth and 10 days later challenged with MOG_35–55_ in CFA and pertussis toxin (MCMV + MOG_35–55_) developed clinical signs that correspond to EAE manifestations seen in C57BL/6 mice (Figure 1A). Based on evaluation of clinical course (Figure 1A) and mean maximal clinical score (Figure 1B), MCMV-infected BALB/c mice developed disease indistinguishable from disease in susceptible C57BL/6 mice. Infiltration in CNS of MCMV + MOG_35–55_, expressed by mean histological score (Figure 1D) and total cell number (Figure 1C), was significantly higher compared with BALB/c mice immunized with MOG_35–55_ only mice. BALB/c mice infected with MCMV and 10 days later immunized with MOG_35–55_ developed subarachnoid and perivascular infiltrations in the brain cortex, perivascular infiltrations in brainstem and cerebellum with spreading to parenchyma (Figure 1E), and white matter spinal cord infiltrations (Figure 1F). Single-cell infiltrates were detected in brains and spinal cords of MOG_35–55_-immunized mice only, and mild perivascular infiltrations were detected in brainstem of MCMV-infected mice (Figures 1E,F). Immunostaining of spinal cord sections showed presence of CD3^+^ cells in the infiltrates (Figure 1G).

### CNS Infiltrates of MCMV + MOG_35–55_ Mice Contain Higher Amounts of T1/T17 CD4^+^ and CD8^+^ T Cells

**Figure 2:**
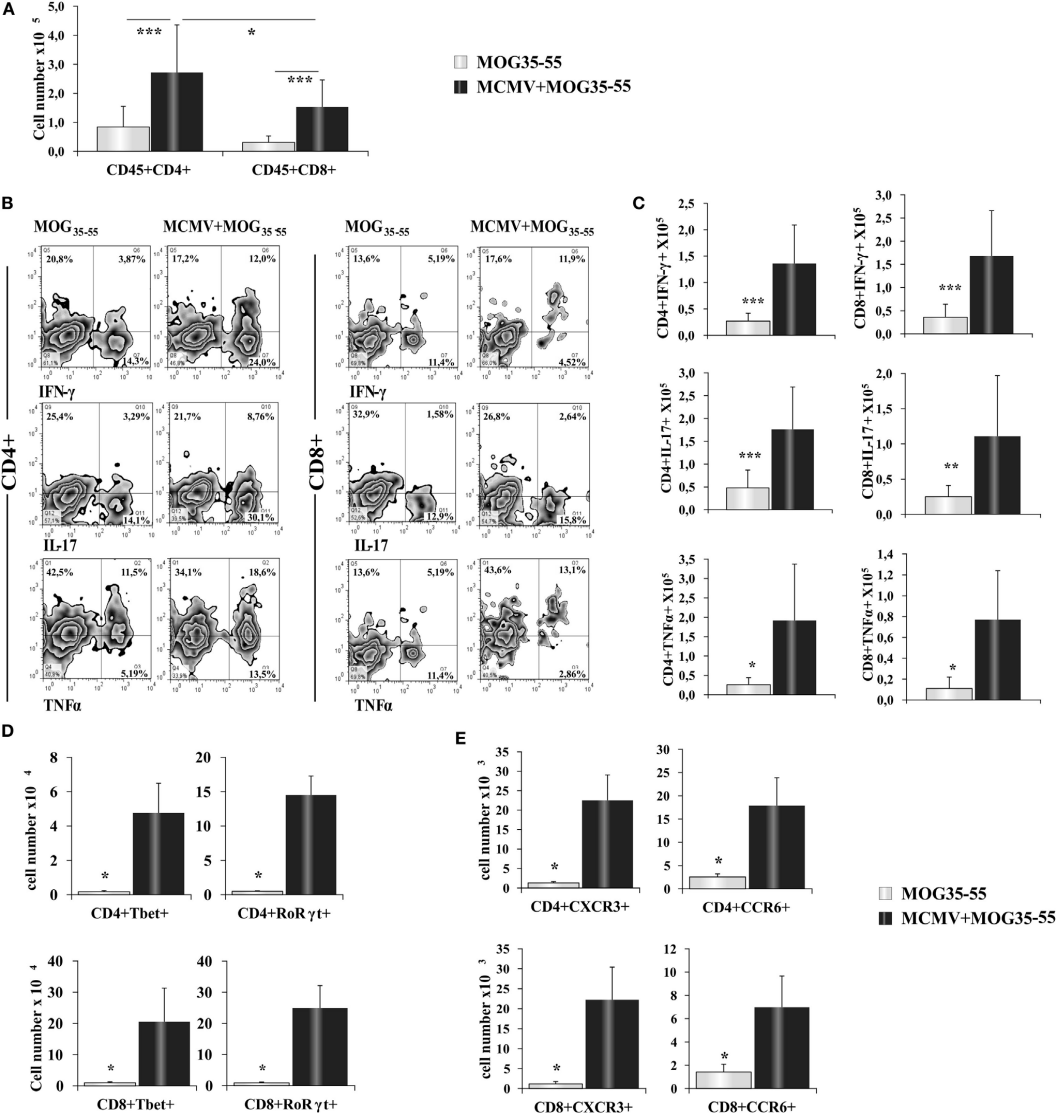
**BALB/c murine cytomegalovirus (MCMV) + MOG_35–55_ mice have increased number of inflammatory CD4^+^ and CD8^+^ cells**. Eight-week-old BALB/c mice were infected with MCMV and 2 weeks after were immunized with MOG_35–55_ peptide. After 15 days, mice were perfused, central nervous system was harvested, and mononuclear cells were isolated and restimulated *ex vivo* with PMA and ionomycin before performing intra cellular staining. **(A)** Total cell numbers of CD45^+^CD4^+^, CD45^+^CD8^+^ cells. **(B)** Representative FACS images of percentages and **(C)** total cell numbers of CD4^+^IFN-γ^+^, CD4^+^TNF-α^+^, CD4^+^IL-17^+^, CD8^+^IFN-γ^+^, CD8^+^TNF-α^+^ and CD8^+^IL-17^+^ cells. Total cell numbers of CD8^+^ cell- and CD4^+^ cell-expressing transcriptional factors T-bet and RORγt **(D)** and chemokine receptors CXCR3 and CCR6 **(E)**. Data from three separate experiments with 22 mice/group are presented as mean + SE. Data were analyzed with Student’s *t*-test: *P < 0.05, **P < 0.005, and ***P < 0.001.

Further analysis showed significantly higher number of CD4^+^ and CD8^+^ T cells in the infiltrates of MCMV + MOG_35–55_ mice compared with MOG_35–55_ mice (Figure 2A). In the CNS of MCMV + MOG_35–55_ mice, there was higher number of CD4^+^ T cells then CD8^+^ T cells, similar to typical EAE in C57BL/6 mice. MCMV + MOG_35–55_ mice had increased percentage (Figure 2B) and number (Figure 2C) of both Th1 and Th17 cells, as well as Tc1 and Tc17 cells compared to MOG_35–55_ mice. Similarly, significantly higher number of CD4^+^ cell- and CD8^+^ cell-expressing transcriptional factors, T-bet and RORγt (Figure 2D), and chemokine receptors CCR6 and CXCR3 (Figure 2E) was noticed in CNS of MCMV + MOG_35–55_ mice compared with MOG_35–55_ mice.

### CD8^+^ Cells Take a Role in Autoimmune Neuropathology in BALB/c Mice with MCMV Infection

**Figure 3:**
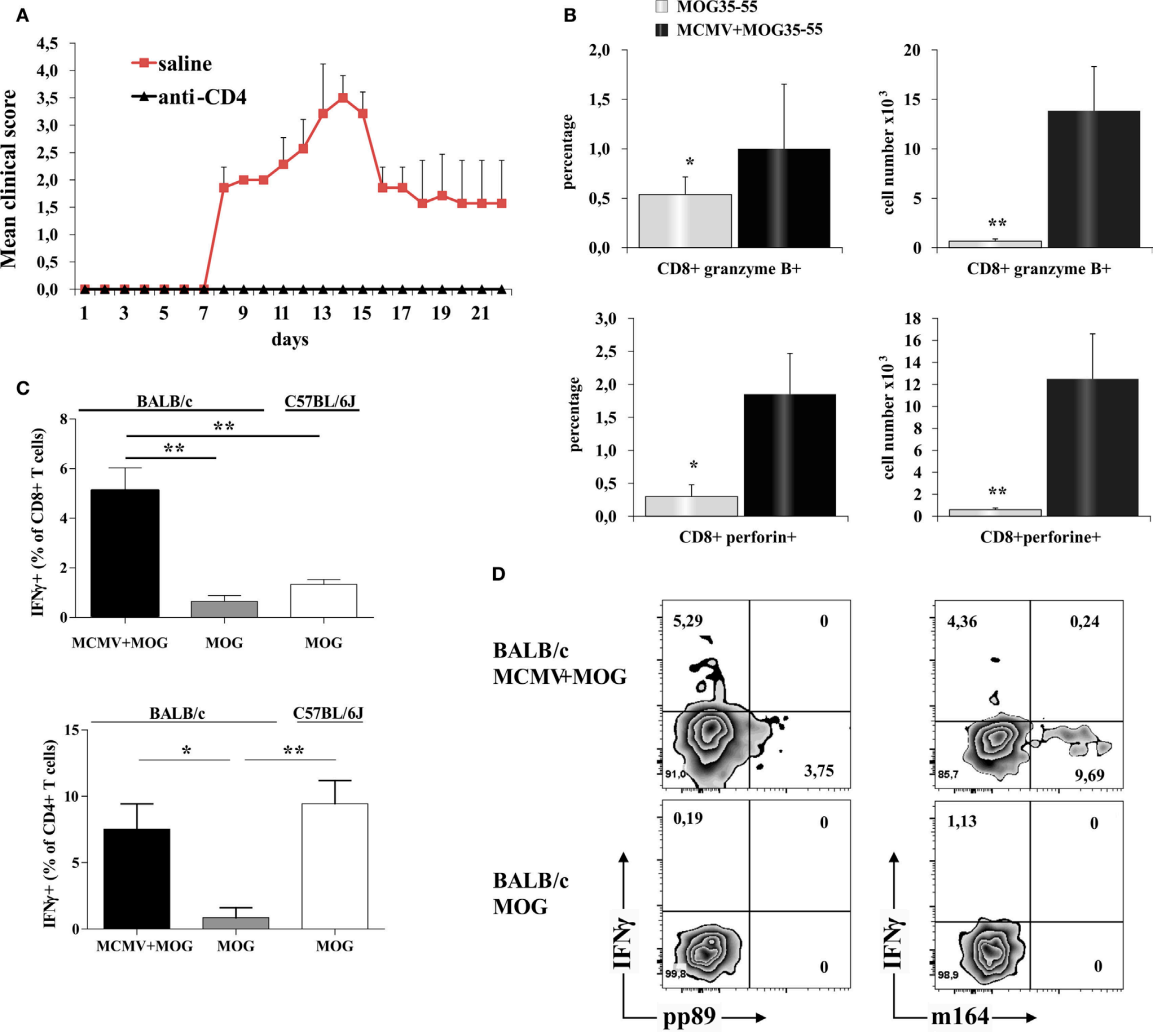
**CD8 T cells in the central nervous system (CNS) of BALB/c murine cytomegalovirus (MCMV) + MOG_35–55_ mice express markers of cytolytic activity and contribute to autoimmune reactions in CNS**. CD4^+^ cells were depleted 10 days after MCMV infection and 5 days before immunization with MOG_35–55_ (anti-CD4), control mice were infected and immunized but received saline instead of depleting antibody (saline). **(A)** Experimental autoimmune encephalomyelitis (EAE) scores up to day 22 post-EAE induction in anti-CD4 and saline mice, data are presented as mean + SE from one experiment with five mice per group. **(B)** Percentages and absolute numbers of CD8^+^granzyme B^+^ and CD8^+^perforin^+^ cells among mononuclear cells isolated from CNS 15 days post-immunization with MOG_35–55_ peptide of BALB/c mice infected with MCMV 10 days earlier (MCMV + MOG_35–55_) and previously untreated BALB/c mice (MOG_35–55_). Data are presented as mean + SE, from two separate experiments with 14 mice/group. **(C)** Percentages of CD4^+^IFN-γ^+^ and CD8^+^IFN-γ^+^ cells and **(D)** representative flow images of IFN-γ, pp89, and m164 expression in CD8^+^ population among mononuclear cells isolated from CNS of MCMV-infected and MOG_35–55_-immunized BALB/c mice and MOG_35–55_-immunized BALB/c and C57BL/6 mice *in vitro* restimulated with MOG_35–55_ peptide. Percentages are presented as mean + SE (representative experiment with six mice per group). Data were analyzed with Student’s *t*-test and Kruskal–Wallis: *P < 0.05, **P < 0.005.

Depletion of CD4^+^ cells in MCMV-infected mice abrogated susceptibility to MOG_35–55_-induced disease (Figure 3A) indicating autoimmune disease. Given the significant number of CD8^+^ cells in the infiltrates that were not seen in classical EAE, we explored in more details these cells found in CNS. There was significantly higher percentage and number of CD8^+^ cell-expressing markers of cytolytic activity in the CNS of MCMV + MOG_35–55_ BALB/c mice compared to MOG_35–55_ mice (Figure 3B). To indirectly determine the percentage of MOG_35–55_-specific CD4^+^ and CD8^+^ cells, mononuclear cells were isolated from CNS of MCMV + MOG_35–55_ BALB/c, MOG_35–55_ BALB/c, and MOG_35–55_ C57BL/6 mice and *ex vivo* restimulated with MOG_35–55_ peptide, and IFN-γ^+^ cells were enumerated. Significantly higher percentage of CD4^+^ and CD8^+^ cells from CNS of MCMV + MOG_35–55_ mice responded to *ex vivo* restimulation compared with MOG_35–55_ only BALB/c mice (Figure 3C). Further, significantly higher percentage of CD8^+^ cells isolated from CNS of MCMV + MOG_35–55_ BALB/c mice contained IFN-γ after *ex vivo* restimulation with MOG_35–55_ compared to C57BL/6 mice immunized with MOG_35–55._ Even more importantly, IFN-γ-containing CD8^+^ cells in MCMV + MOG_35–55_ BALB/c mice after restimulation with MOG_35–55_ were not specific for viral epitopes pp89 and m164, suggesting that inflammatory CD8^+^ cells in the CNS are autoimmune (Figure 3D).

### Chronic Non-Productive MCMV Infection Also Facilitates EAE Development in BALB/c Mice

**Figure 4:**
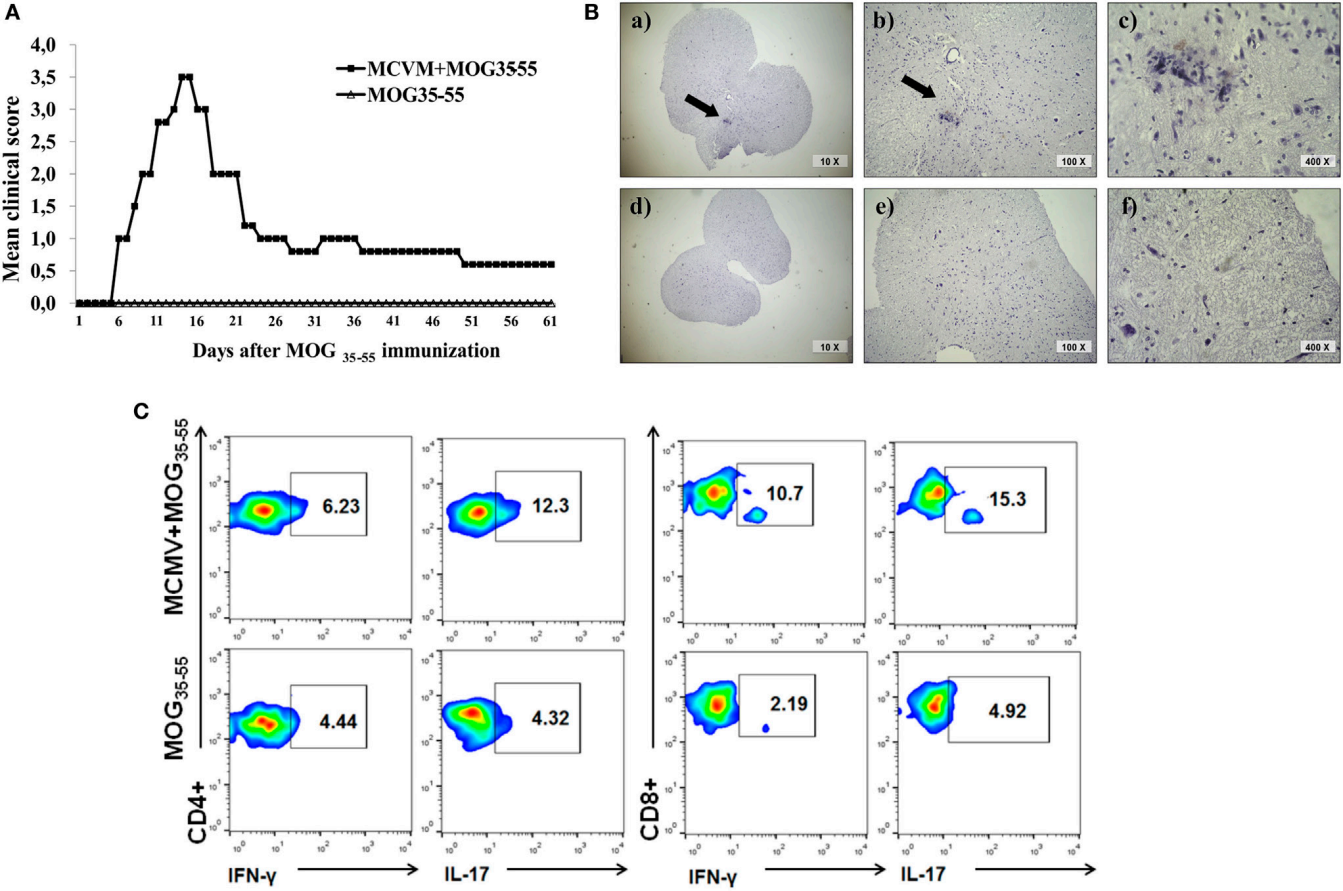
**BALB/c mice with latent murine cytomegalovirus (MCMV) infection develop experimental autoimmune encephalomyelitis and longlasting infiltrates in central nervous system**. Mice were infected with MCMV, and 3 months later they were immunized with MOG_35–55_ peptide, and disease was evaluated for 60 days. **(A)** Mean clinical score and **(B)** representative images of spinal cord sections 60 days after immunization with MOG_35–55_ peptide in MCMV + MOG_35–55_ group (a–c) and MOG_35–55_ group (d–f). **(C)** Representative flow cytometric images presenting percentages of IL-17- and IFN-γ-expressing CD4^+^ and CD8^+^ cells among mononuclear cells isolated from brains 60 days after immunization with MOG_35–55_ peptide. Presented data are from representative experiment with seven mice per group.

In order to test whether the chronic non-productive MCMV infection could facilitate EAE development, we immunized BALB/c mice with MOG_35–55_ peptide 3 months after MCMV infection in adult life. As shown in Figure 4, infected mice developed clinical signs of EAE while age-matched mice immunized with encephalitogen only, did not. Mice with chronic non-productive MCMV infection started to manifest signs of EAE 6 days after immunization with MOG_35–55_ peptide; maximal clinical score reached 15 days after immunization and had very mild signs of the disease 60 days after immunization (Figure 4A). Chronic disease was confirmed with histological analysis. Perivascular infiltrates were detected in the spinal cords of mice with latent MCMV infection 2 months after challenge with MOG_35–55_ peptide (Figure 4B). Among cells isolated from brains of MCMV-infected BALB/c mice 2 months after MOG_35–55_ immunization, higher percentage of CD4^+^ and CD8^+^ cells contained inflammatory cytokines IL-17 and IFN-γ, after *in vitro* restimulation with MOG_35–55_ peptide compared to stimulated cells isolated from MOG_35–55_-immunized mice (Figure 4C). Our findings indicate that BALB/c mice with latent MCMV infection develop disease with long-lasting infiltrates in the CNS that contains lymphocytes specific for neuroantigens.

### MCMV Infection Induces Inflammatory Phenotype of Antigen-Presenting Cells in Periphery and in CNS

**Figure 5:**
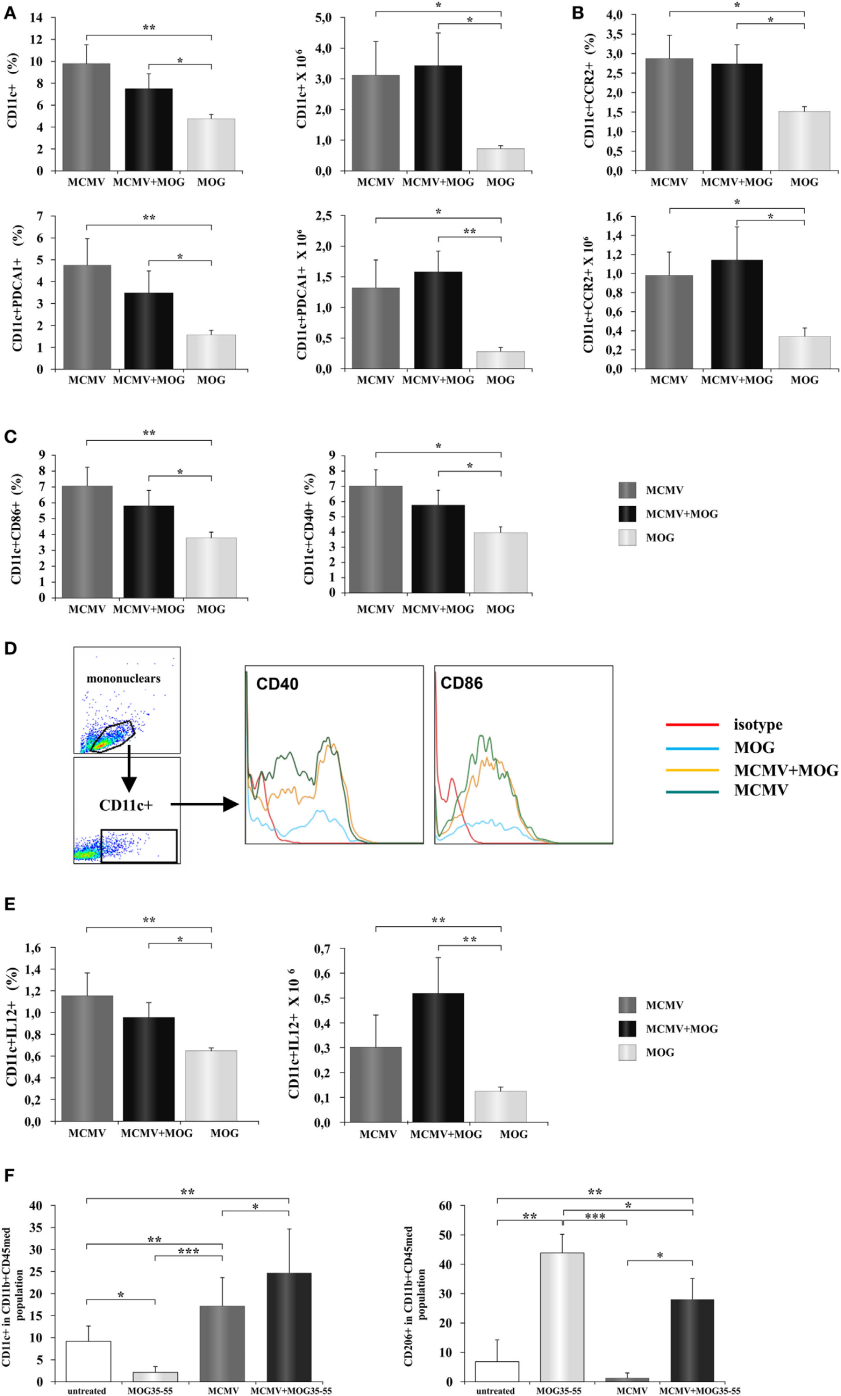
**Murine cytomegalovirus (MCMV) infection favors inflammatory phenotype of antigen-presenting cells**. Mononuclear cells were isolated from inguinal lymph nodes 2 days after immunization with MOG_35–55_ peptide (MOG_35–55_) and from lymph nodes of mice 10 days after their MCMV infection (MCMV). Flow cytometric analysis of dendritic cells phenotype was done. **(A)** Percentages and absolute numbers of CD11c^+^ dendritic cells and CD11c^+^CD11b^−^PDCA1^+^ dendritic cells, **(B)** CCR2-expressing CD11c^+^ cells, and **(C)** CD86^+^ and CD40^+^ dendritic cells are presented as mean + SE (10 mice per group). **(D)** Representative histograms of CD40 and CD86 expression in CD11c^+^ population. **(E)** Percentages and absolute numbers of IL-12^+^ dendritic cells presented as mean + SE (10 mice per group). **(F)** Mononuclear cells were isolated from central nervous system of saline-treated, MOG_35–55_-immunized, MCMV-infected, and MCMV-infected and MOG_35–55_-immunized mice, 15 days after MOG_35–55_ immunization percentages of classically (CD11c^+^) and alternatively (CD206^+^) microglia are presented as mean + SE (8–10 mice per group). Data were analyzed with Student’s *t*-test and ANOVA: *P < 0.05, **P < 0.005, and ***P < 0.001.

It is known that viral infection induces antiviral immune response mediated by NK cells, CD8^+^ and CD4^+^ lymphocytes (33). Such inflammatory microenvironment in peripheral lymph organs can affect activation of antigen-presenting cells and thus indirectly contribute to development of inflammatory lymphocytes. Therefore, we explored possible influence of viral infection on changes of phenotype of antigen-presenting cells. To this end, mononuclear cells were isolated from inguinal lymph node 12 days after MCMV was administered in foot pad and compared with cells isolated from mice immunized with MOG_35–55_ only. Lymph nodes of MCMV-infected and MCMV + MOG_35–55_ mice had significantly higher percentage of CD11c^+^ dendritic cells and CD11c^+^PDCA1^+^plasmocitoid dendritic cells in inguinal lymph nodes compared to MOG_35–55_-immunized mice (Figure 5A). Also higher percentage of dendritic cell-expressing CCR2 chemokine receptor was found in both groups of MCMV-infected mice (Figure 5B). More importantly, lymph nodes of MCMV-infected and MCMV + MOG_35–55_ mice contained higher percentage of activated CD86^+^ and CD40^+^ (Figure 5C) and inflammatory IL-12^+^ dendritic cells (Figure 5E). Higher expression of markers of activation, CD86^+^ and CD40^+^ was noticed in MCMV-infected mice compared with MOG_35–55_-immunized mice (Figure 5D). These data suggest that inflammatory phenotype of dendritic cells is achieved in BALB/c mice with viral infection but not with encephalitogenic challenge only, as it was seen in C57BL/6 mice.

It is known that systemic inflammation in mice causes activation of microglia that persists for months (34, 35). Since MCMV infection in BALB/c mice causes systemic inflammation, we wanted to see effect of MCMV infection on phenotype of antigen-presenting cells in CNS. We analyzed expression of markers of classical (CD11c) and alternative activation (CD206) in the population of microglia (CD45^int^CD11b^+^) in healthy mice, MOG_35–55_-immunized mice, MCMV-infected, and MCMV + MOG_35–55_ mice. As shown in Figure 5F, microglia of mice with viral infection in adult life, with and without EAE, had proinflammatory, M1 phenotype. Significantly higher percentage of CD11c-expressing cells in microglia population was found in both groups of infected mice compared to healthy and MOG_35–55_-immunized mice. On the other hand, the highest percentage of (type 2) CD206-expressing microglia was found in MOG_35–55_ BALB/c mice. Higher percentage of M2 microglia was also found in MCMV + MOG_35–55_-immunized mice compared to healthy and MCMV-infected mice but lower compared to MOG_35–55_-immunized mice. Thus, high percentage of M2 microglia in mice at the peak of EAE could be also the compensatory mechanism that precedes disease attenuation.

## Discussion

Here, we provide the first evidence that MCMV infection results in breaking resistance of BALB/c mice to EAE induction with MOG_35–55_ peptide, as indicated by typical clinical manifestations and massive inflammatory infiltration in the CNS (Figure 1).

The role of MCMV infection in EAE is not studied, while its significance in MS is controversial. There are several prospective clinical studies that indicate protective effect of CMV infection on MS risk (21, 22, 36), while recent well-powered meta-analysis found no significant difference in the rate of CMV seropositivity between MS patients and healthy controls based on pooled samples from all studies to date (37). On the other hand, CMV has been found in demyelinating plaques and the liquor of MS patients (15), and several clinical studies support the role of CMV in MS pathogenesis (18–20). Additionally, CMV infection induces expansion of inflammation-seeking/proinflammatory effector-memory CD4^+^CD28^null^ T cells that are attracted to MS lesions *via* a CX3CL1 gradient (38, 39) and are mostly found in MS patients (40). Stimulation of these cells with myelin autoantigens results in their proliferation and release of cytotoxic granules, and thus may contribute to MS pathology (41). Some experimental animal studies support the role of CMV in EAE development. Cross-reactivity between CMV_981–1,003_ and MOG_35–55_ peptides was found in Lewis rats immunized with MOG_35–55_ (42), while cross-reactivity between CMV_981–1,003_ and MOG_34–56_ was found in rhesus monkeys (43). Immunization of rhesus monkeys with human CMV_981–1,003_ peptide induced expansion of MOG_34–56_-specific T cells (43). Female SJL/J mice primed with vaccinia virus that contain PLP gene and later challenged with MCMV-developed lesions in white matter regions in the brains (28). Other studies on primates also support the role of CMV in MS pathogenesis (44, 45). Recently, Juranic Lisnic et al. demonstrated that MCMV infection of murine fibroblasts induced highest expression of interferon β, transcriptional factor T-bet, chemokine CXCL10 (46), and the role of these markers of Th1 cells, in EAE pathogenesis is well known (47–49).

However, there was no evidence that MCMV infection may directly facilitate EAE. Here, we show that adult MCMV infection overcomes resistance of BALB/c mice to induction of EAE with MOG_35–55_ peptide. The disease is characterized by typical clinical signs (Figure 1A) seen in susceptible C57BL/6 mice and massive brain and spinal cord infiltrations (Figures 1E,F). It should be noted that brain infiltrates are more significant in BALB/c mice treated with encephalitogen then in “classical” EAE in C57BL/6 mice. It appears that the disease after infection + MOG_35–55_ challenge in otherwise resistant mice is more similar to MS than classical EAE (50). While in C57BL/6 mice, CD4^+^ cells dominate in CNS infiltrates in the experiments presented here, there was similar number of CD4^+^ and CD8^+^ cells in the infiltrates (Figure 1).

Encephalitogenity of CD4^+^ T cells in the infiltrates in BALB/c mice is further documented by their expression of chemokine receptors CXCR3 (Figure 2E), whose blockade during EAE induction attenuated the disease (51), and CCR6 receptor known to have the key role in development of initial autoimmune infiltration in the CNS (52, 53). While γHV-68 infection in C57BL/6 mice with EAE leads to almost exclusive infiltration with Th1 cells (7), in the CNS of MCMV-infected BALB/c mouse with EAE there is almost equal participation of Th1 and Th17 cell-expressing IFN-γ and T-bet (Th1) and IL-17 and RORγt (Th17) (Figures 2C,D). Moreover, inflammatory infiltrates in MCMV-pretreated BALB/c mice immunized with MOG_35–55_ contained CD8^+^ cell-expressing T1 and T17 transcriptional factors and corresponding cytokines TNF-α and IFN-γ (Tc1) and IL-17 (Tc17 cells) (Figures 2C,D). Interestingly, it was suggested that Tc17 cells are required for Th17 accumulation and development of MS (54). Patients with early-stage MS harbor a greater number of Tc17 cells in the cerebrospinal fluid than in peripheral blood that contribute to the initiation of CNS autoimmunity (54). Since in the CNS of BALB/c mice with neonatal MCMV infection but without immunization with MOG_35–55_ dominates Tc1 cells (IFN-γ and T-bet^+^) (55), it appears that the newly developing autoimmune process attracts a different population of CD8^+^ cells (IL-17 producing) that contribute to autoimmune process (54).

Significant accumulation of CD8^+^ cells in the infiltrates and their role in autoimmune pathogenesis is documented by clinical signs and typical pathology complemented by specificity of infiltrating cells. *Ex vivo* restimulation with MOG_35–55_ leads to significant increase of CD8^+^ cell-producing IFN-γ in MCMV + MOG_35–55_ BALB/c mice (Figure 3C). This finding is at variance with our finding in C57BL/6 mice. CD8^+^ cells from EAE C57BL/6 mice did not responded to restimulation with MOG_35–55_ (Figure 3C)_._ Further, MOG_35–55_ responsive CD8^+^ cells in MCMV + MOG_35–55_ BALB/c mice were not specific for viral epitopes pp89 and m164 (Figure 3D) implicating that CD8^+^ cell contributes to autoimmune process in this model of EAE. This finding is in line with previous report that initiation of autoimmune process in CNS with CD4^+^ T cells is followed with spreading to myelin-specific CD8^+^ T cells that are capable of direct recognition of oligodendrocytes and contribute to tissue damage (56). Similarly, heighten EAE in C57BL/6 mice infected with γHV-68 is accompanied with infiltration of brain parenchyma with CD8^+^IFN-γ^+^granzyme^+^ cells (7). However, specificity for autoantigen of these inflammatory and cytolytic CD8^+^ cells in γHV-68-infected C57BL/6 mice was not studied (7). Finding that mice with depletion of CD4^+^ cells after MCMV infection and before MOG_35–55_ immunization did not develop the disease (Figure 3A) contributes to the conclusion that MCMV-infected BALB/c mice developed autoimmune neuropathology. Persistence of CNS infiltrations and MOG_35–55_-specific CD4^+^ and CD8^+^ cells in CNS of BALB/c mice 2 months after MOG_35–55_ immunization that were infected 3 months previously (Figure 4) also proving autoimmune nature of the disease. Thus although CD4^+^ cells are required, it appears that CD8^+^ cells are the main effector cells.

MCMV infection significantly increases proportion of dendritic cells (CD11c^+^), plasmocitoid dendritic cells (CD11c^+^PDCA1^+^) in peripheral lymph nodes (Figure 5A) compared to immunization with MOG_35–55_. Further higher percentage of dendritic cells in lymph nodes in MCMV-infected mice is accompanied with higher percentage of CCR2^+^ dendritic cells (Figure 5B). It is known that MCMV encodes proinflammatory factor (MCK-2), analog of chemokine CCL2 (57) that enhances monocyte recruitment and viral dissemination (58). Then, higher percentage of dendritic cells in inguinal lymph nodes of MCMV-infected mice could be the consequence of CCL2 analog production, since CCL2 goes to lymph nodes where is presented on the surface of high endothelial venules for recruitment of monocytes (59). Although it was previously shown that MCMV attracts monocytes that have the immunosuppressive role (60), here, we found higher percentage of dendritic cell-expressing markers of activation CD86 and CD40 (Figure 5C) and containing Th1 promoting cytokine, IL-12 (Figures 5D,E). Our results indicate that MCMV infection of BALB/c mice induces increase of inflammatory dendritic cells in peripheral lymph nodes and thus enables development of encephalitogenic T cells. This finding is in correlation with previous report that MCMV-infected mice are resistant to bacterial infection due to prolonged production of the antiviral cytokine IFN-γ and systemic activation of macrophages (61). Significantly, there was an increase of classically activated microglia (CD45^med^CD11b^+^CD11c^+^) in the CNS of BALB/c mice 25 days after MCMV infection compared to MOG_35–55_-immunized mice without previous infection that had mostly alternatively activated microglia (CD45^med^CD11b^+^CD206^+^) (Figure 5F). Lower percentage of alternatively activated microglia in MCMV + MOG_35–55_ mice observed on day 15 after immunization and day 25 after infection in compared to MOG_35–55_ treated mice, and prevalence of M1 microglia in virus-infected mice may contribute to chronic disease in MCMV-infected MOG_35–55_-immunized BALB/c mice. Previously, it was shown that systemic MCMV infection elicited a significant increase in the number of microglia with morphological signs of activation and M1 phenotype (62). Thus, it appears that at least one level of resistance of BALB/c mice to EAE is the inability to convert microglia into M1 phenotype.

In summing up, we report here that MCMV infection may promote autoimmune neuropathology and convert resistant mice into susceptible to EAE induction. This was achieved by activation of antigen-presenting cells and promoting M1 phenotype of microglia as well as participation of CD8^+^ encephalitogen-specific T cells in the autoimmune pathogenesis.

## Author Contributions

Conceived and designed the experiments: JM, MM, NA, SJ, and ML. Performed the experiments: JM, BP, MM, AA, BS, and DK. Analyzed the data: JM, BP, MM, DK, IT, and AK. Wrote the paper: JM and ML.

## Conflict of Interest Statement

The authors declare that the research was conducted in the absence of any commercial or financial relationships that could be construed as a potential conflict of interest.
